# Otic Langerhans' Cell Histiocytosis in an Adult: A Case Report and Review of the Literature

**DOI:** 10.1155/2013/259726

**Published:** 2013-05-23

**Authors:** Anil Gungadeen, Peter Kullar, Philip Yates

**Affiliations:** ENT Department, Freeman Hospital, Newcastle upon Tyne NHS Foundation Trust, Newcastle upon Tyne NE7 7DN, UK

## Abstract

*Objective*. To present a case of otic Langerhans' cell histiocytosis in an adult. Also included the diagnosis and management of the condition and a review of the relevant literature. *Case Report*. We report a case of a 41-year-old man with a history of persistent unilateral ear discharge associated with an aural polyp. Radiological imaging showed bony lesions of the skull and a soft-tissue mass within the middle ear. Histological analysis of the polyp demonstrated Langerhans' cell histiocytosis. His otological symptoms were completely resolved with the systemic therapy. *Conclusions*. Otic Langerhans' cell histiocytosis can present in adults. Persistent ear symptoms along with evidence of soft-tissue masses within the ear and bony lesions of the skull or elsewhere should prompt the otolaryngologists to include Langerhans' cell histiocytosis in their differential diagnosis. Management should be with systemic therapy rather than local surgical treatment.

## 1. Introduction

Langerhans' cell histiocytosis (LCH) is a rare disease that is characterised by the idiopathic proliferation of abnormal histiocytes, either in one organ or as part of a systemic disease [1–3]. LCH is predominantly known to be a disease affecting children and is rare in adults. LCH has been reported in paediatric studies to involve the ear and temporal bones in 14% to 61% of cases [4]. However, only a few cases of ear involvement have been reported in adults. This report describes a case of LCH affecting the ear in a 41-year-old patients and it is our recommendation that nonsurgical management is preferable.

## 2. Case Report

A 41-year-old self-employed brewery engineer was referred to the ENT department with a four-week history of persistent left-sided mucopurulent ear discharge, despite topical antibiotic therapy including gentamicin. He did not have any other otological symptoms, nor any rhinological or upper aerodigestive tract symptoms. His hearing was within normal limits. He was a smoker of 30 cigarettes per day, had a past medical history of hypertension, and had been recently diagnosed with Langerhans' cell histiocytosis involving the skull, for which he was under the care of Haematology. His medication list included azathioprine for LCH, antihypertensives, and a statin.

On examination, his left external auditory canal was very oedematous and was filled with an inflammatory polyp. Cranial nerve examination was unremarkable.

**Figure 1:**
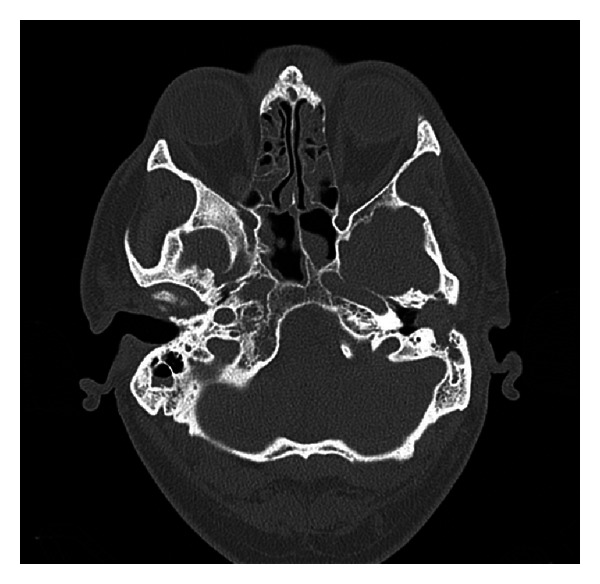
CT petrous bones axial image showing left middle ear soft-tissue mass.

He had a PET-CT as part of his LCH workup, which showed increased fludeoxyglucose (FDG) uptake in the frontal bone, left parietal bone, right occipital bone, and right sphenoid wing, as well as a 1.7 cm mass within the left middle ear. He also subsequently had a CT of his petrous temporal bones, as represented in Figure 1, which showed a 1.7 × 1.4 cm expansile soft-tissue mass within the lateral aspect of left middle ear and superior mastoid, prolapsing into external ear canal and associated with destruction of tegmen tympani, scutum, and the roof of the external ear canal; the mass was also seen to abut onto the ossicles but did not cause any ossicular erosion. The right petrous temporal bone was unremarkable.

**Figure 2:**
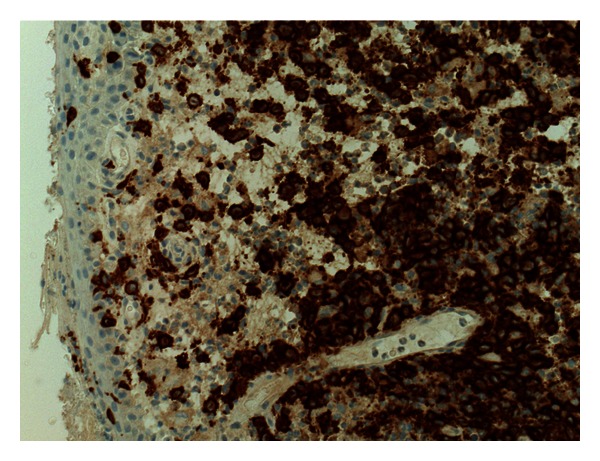
CD1a immunohistochemical stain highlighting the Langerhans cells (×20).

The patient underwent an examination under anaesthesia of his left ear which demonstrated a polyp arising from the bulging posterior extent of the external auditory canal. The polyp was excised and sent for histological analysis. The stroma contained granulation tissue and a dense mixed inflammatory cell infiltrate which included abundant eosinophils and numerous Langerhans' cells. Immunohistochemically, CD68 highlighted numerous macrophages and Langerhans' cells. Langerhans' cells are also labelled with S100 and CD1a (Figure 2). These appearances were consistent with Langerhans' cell histiocytosis. 

Although he was asymptomatic with regards to his skull lesions, the persistence of otorrhoea from the affected ear indicated continued LCH activity. The dose of azathioprine was therefore doubled from 50 mg to 100 mg, following which his otorrhoea resolved. After his ear symptoms remained under control for more than six months, he was discharged from ENT followup and remained under the care of Haematology for regular monitoring of the LCH. He has not required any further ontological input.

## 3. Discussion

LCH is predominantly a disease of childhood and has been well documented in the paediatric population, with a mean age at presentation of three years [2, 5, 6]. McCaffrey et al. in their study of 22 patients in 1979 suggested, however, that LCH might have a secondary peak incidence between 30 and 39 years of age [7]. The report from the International Registry of the Histiocyte Society published by Aricò et al. in 2003 shows the mean age of diagnosis of LCH in adults to be 35 years [8].

Ear and temporal bone involvement occurs in 14 to 61% of children with LCH [7], but only few cases have been reported in adults [7, 9–11]. Ear involvement can be in the form of chronic otitis externa or otitis media, external ear canal mass or polyp, postauricular swelling, conductive hearing loss and rarely facial paralysis, and vertigo [2, 12]. Involvement can be bilateral in 30% of patients [2] and is more likely to occur as part of multifocal or systemic LCH [3, 12]. Up to 25% of patients are reported to present with ear involvement as their only symptoms [13].

This case demonstrates that persistent ear discharge in adults can be due to LCH. In such a patient presenting to the otolaryngologist, with radiological evidence of a soft-tissue mass within the middle ear and external ear canal with or without bony erosion and other skull lesions, the diagnosis of LCH should be considered. CT is useful in delineating bony involvement and shows bony destruction of petrous apex, and MRI gives information about the extent of any soft-tissue involvement [13]. Bone scintigraphy can also reveal other lesions, particularly in complex bones [13]. The typical radiological appearance of LCH is a shining lesion, with sclerotic margins and a bevelled edge, with homogeneous soft-tissue masses enhancing uniformly with administration of intravenous contrast [4, 13]. Tissue diagnosis is the gold standard to confirm the diagnosis, with immunohistochemistry showing Langerhans' cells associated with an inflammatory infiltrate consisting of lymphocytes, plasma cells, giant cells, and large numbers of eosinophils [4].

Treatment of LCH depends on the pattern of the disease. Localised disease may be treated with surgical excision and intralesional steroids [1, 2, 5, 6], whereas a more conservative approach is advocated for systemic disease. Immunosuppression has been used to treat systemic LCH, but the limited number of studies in which it has been reported makes analysis of its efficacy difficult [1]. The mainstay of treatment of systemic disease is chemotherapy, with vinblastine commonly used in combination with a steroid [1].

The majority of patients presenting with ear involvement, as discussed above, suffer from systemic LCH. It is our recommendation that otic LCH should not be managed surgically. This is due to the extreme difficulty in resecting diseased tissue *in toto* with the concomitant risks of serious complications such as conductive and sensorineural hearing loss, facial nerve paralysis, postoperative fistula, and persistent activity of the disease [6, 9]. 

## 4. Conclusion

Although Langerhans' cell histiocytosis is a disease known to predominantly affect children, adults can also be affected, including within the ear and temporal bone. Nonresolving ear symptoms along with evidence of soft-tissue masses within the ear and bony lesions of the skull or elsewhere should prompt the otolaryngologists to include LCH in their differential diagnosis. Once tissue diagnosis has been confirmed, treatment should be systemic rather than surgical.
